# MicroRNA Biomarker *hsa-miR-195-5p* for Detecting the Risk of Lung Cancer

**DOI:** 10.1155/2020/7415909

**Published:** 2020-01-02

**Authors:** Lei Li, Tienan Feng, Weituo Zhang, Sumeng Gao, Ruoyang Wang, Wenwen Lv, Tengteng Zhu, Herbert Yu, Biyun Qian

**Affiliations:** ^1^Hongqiao International Institute of Medicine, Shanghai Tongren Hospital and Faculty of Public Health, Shanghai Jiao Tong University School of Medicine, Shanghai, China; ^2^Clinical Research Center, Shanghai Jiao Tong University School of Medicine, Shanghai, China; ^3^Hongqiao International Institute of Medicine, Shanghai Tongren Hospital and Clinical Research Institute, Shanghai Jiao Tong University School of Medicine, Shanghai, China; ^4^Cancer Epidemiology Program, University of Hawaii Cancer Center, Honolulu, HI, USA

## Abstract

**Background:**

Lung cancer is one of the leading diagnosed cancers worldwide, and microRNAs could be used as biomarkers to diagnose lung cancer. *hsa-miR-195* has been demonstrated to affect the prognosis of NSCLC (non-small-cell lung cancer) in a previous study. However, the diagnostic value of *hsa-miR-195-5p* in lung cancer has not been investigated.

**Methods:**

To evaluate the ability of *hsa-miR-195-5p* to diagnose lung cancer, we compared the expression of *hsa-miR-195-5p* in lung cancer patients, COPD patients, and normal controls. Receiver operating characteristic (ROC) curve analysis was performed to investigate the sensitivity and specificity of *hsa-miR-195-5p*. Coexpression network and pathway analysis were carried out to explore the mechanism.

**Results:**

We found that *hsa-miR-195-5p* had lower expression in lung cancer and COPD patients than in normal controls, and the AUC was 0.92 for diagnosing lung cancer. *hsa-miR-143* correlated with *hsa-miR-195-5p*, and by combining these two microRNAs, the AUC was 0.97 for diagnosing lung cancer.

**Conclusions:**

*hsa-miR-195-5p* may act as a biomarker that contributes to the diagnosis of lung cancer and the detection of its high-risk population.

## 1. Background

Lung cancer is one of the leading diagnosed tumors with high mortality worldwide [1] and China [2]. It is estimated 2.09 million new lung cancer cases and 1.96 million lung cancer deaths worldwide in 2018 [1]. The 5-year survival estimates in lung cancer range from 73% in stage IA to 13% in stage IV [3]. Unfortunately, around 80% of patients with lung cancer have stage III or IV disease at presentation [4]. Surgery, radiotherapy, chemotherapy, target therapy, and immunotherapy significantly improve the survival and quality of life of lung cancer patients [5], especially in early-stage lung cancer [6]. However, only some subsets of patients in certain tumor types are suitable for target therapy [7] and drug resistance remains a big challenge [8]. Early diagnosis and detection of lung cancer are effective strategies for prevention and treatment [9]. Low-dose computed tomography is a common and effective early screening method for lung cancer [10]. However, lung cancer screening with low-dose computed tomography has some limitations including increased costs, high rate of nodule detection, overdiagnosis [11], and radiation exposure [12]. It is accepted that biomarkers for early diagnosis could help reduce mortality for lung cancer [13]. The identification of genomic biomarkers such as the epidermal growth factor receptor (EGFR) and anaplastic lymphoma kinase (ALK) has improved the current clinical practice [14]. Despite most published guidelines relating to the diagnosis and management of patients with lung cancer do not recommend any serum biomarker, serum biomarker assays are performed in some European and Asian countries [15]. A number of diagnostic biomarkers for lung cancer have been suggested, including carcinoembryonic antigen (CEA), neuron-specific enolase (NSE), cytokeratin 19 (CYFRA-21-1), alpha-fetoprotein (AFP), serum carbohydrateantigen-125 (CA-125), carbohydrate antigen-19.9 (CA-19.9), and ferritin [14, 16]. Biomarkers as a safe and efficient way to combine with low-dose computed tomography and other methods can improve the early diagnosis of lung cancer [17]. COPD (chronic obstructive pulmonary disease) shares many high-risk factors with lung cancer, and COPD itself is a risk factor for the development of lung cancer [18].

In lung cancer, many biomarkers have been discovered and reported to predict lung cancer risk or diagnose lung cancer. Epigenetic changes, protein and proteomic signatures, gene mutations, RNA expression levels, and loss of gene heterozygosity all can serve as biomarkers of lung cancer [19]. Of these biomarkers, microRNAs are a class of short RNAs that regulate gene expression and have been widely investigated as potential biomarkers in lung cancer [20].

In a previous study from our research group [21], we found that microRNA *hsa-miR-195-5p* suppresses NSCLC (non-small-cell lung cancer) and predicts lung cancer prognosis. In addition to our research, previous studies have shown aberrant *hsa-miR-195-5p* expression in multiple cancer types, such as prostate cancer [22], hepatocellular carcinoma [22], and cervical cancer [23]. However, the expression level of *hsa-miR-195-5p* in normal controls, lung cancer patients, and COPD patients, who have a high risk for developing lung cancer, has not been investigated, and the diagnostic ability of *hsa-miR-195-5p* in lung cancer has not been evaluated. Therefore, we carried out this study to investigate these factors.

## 2. Methods

### 2.1. Data Source

All datasets were obtained from GEO (Gene Expression Omnibus) [24] and TCGA (The Cancer Genome Atlas) [25] databases with open access. We searched for datasets that had at least two types of people, either lung cancer or COPD patients and normal controls, in the GEO database. We selected datasets that had more than thirty participants in the study, for the next step of the analysis. All the GEO datasets contained noncoding RNA profiling by array using different platforms. The TCGA database had LUAD (lung adenocarcinoma) and LUSC (lung squamous cell carcinoma) microRNA data. The LUAD dataset had 46 normal controls and 456 lung cancer patients. The LUSC dataset had 45 normal controls and 342 lung cancer patients. We also combined mRNA data of TCGA-LUAD and TCGA-LUSC in a coexpression analysis. The details of these datasets are summarized in [Supplementary-material supplementary-material-1].

### 2.2. Coexpression Network

We merged microRNA and mRNA data from the TCGA data and combined the LUAD and the LUSC samples. Then, we calculated a correlation matrix based on the Pearson correlation coefficient. We selected microRNAs directly linked with the *hsa-miR-195-5p* microRNA with cutoffs of *R*^2^ > 0.5 and *p* < 0.05. To determine other microRNAs that were indirectly linked with *hsa-miR-195-5p* in the network, we set cutoffs of *R*^2^ > 0.7 and *p* < 0.05 between indirectly linked microRNAs. We used a network diagram to show this coexpression network.

### 2.3. Statistical Analyses

All datasets were normalized using zero-mean normalization. A *t*-test was used to evaluate different expressions between different types of samples, and a *p* value < 0.05 was considered statistically significant. When showing the expression of *hsa-miR-195-5p* in the bar chart, the expression of the control group was set to one and the fold changes of other groups were calculated. Receiver operating characteristic (ROC) curves and area under the curve (AUC) were conducted to evaluate the ability of biomarkers to distinguish between lung cancer or COPD patients and normal controls. All statistics were performed using R software (version 3.4.1, URL: https://cran.r-project.org/bin/windows/base/old/3.4.1/).

### 2.4. Pathway Analysis

We uploaded the microRNA data from the coexpression network to IPA (Ingenuity Pathway Analysis, URL: https://www.qiagenbioinformatics.com/products/ingenuity-pathway-analysis/) to explore the mechanism and function of miRNAs. In the pathway enrichment and function analysis, we selected significant pathways and functions depending on *p* < 0.05.

## 3. Results

### 3.1. The Expression of *hsa-miR-195-5p* in Lung Cancer Patients, COPD Patients, and Normal Controls

**Figure 1:**
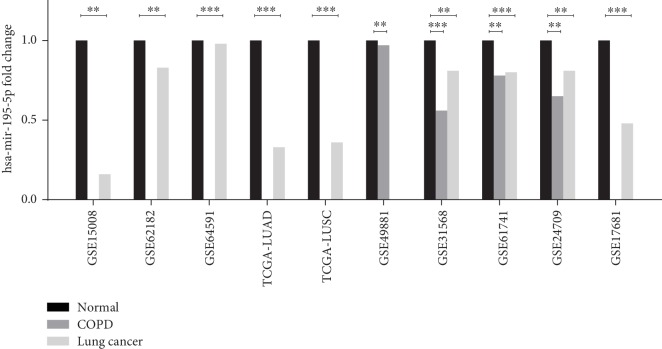
Expression of *hsa-miR-195-5p* in lung cancer patients, COPD patients, and normal controls. All datasets show higher expression of *hsa-miR-195-5p* in lung cancer and COPD patients compared with normal controls. Because of the absence of samples, some datasets do not contain all three population types.

Among the datasets, GSE15008, GSE62186, GSE64519, TCGA-LUAD, TCGA-LUSC, and GSE17681, which contained lung cancer patients and normal controls, *hsa-miR-195-5p* showed lower expression in lung cancer patients compared with normal controls (*p* < 0.05). In the dataset GSE49881, which had COPD patients and normal controls, *hsa-miR-195-5p* presented lower expression in COPD patients compared with normal controls (*p* < 0.05). The GSE31568, GSE61741, and GSE24709 datasets contained lung cancer patients, COPD patients, and normal controls, among which lung cancer and COPD patients had lower expression of *hsa-miR-195-5p* than normal controls (*p* < 0.05). However, there were no differences between lung cancer and COPD patients in *hsa-miR-195-5p* expression.  Figure 1 shows the details of these analyses.

### 3.2. The Association of *hsa-miR-195-5p* with Smoking Status and Sex

**Figure 2:**
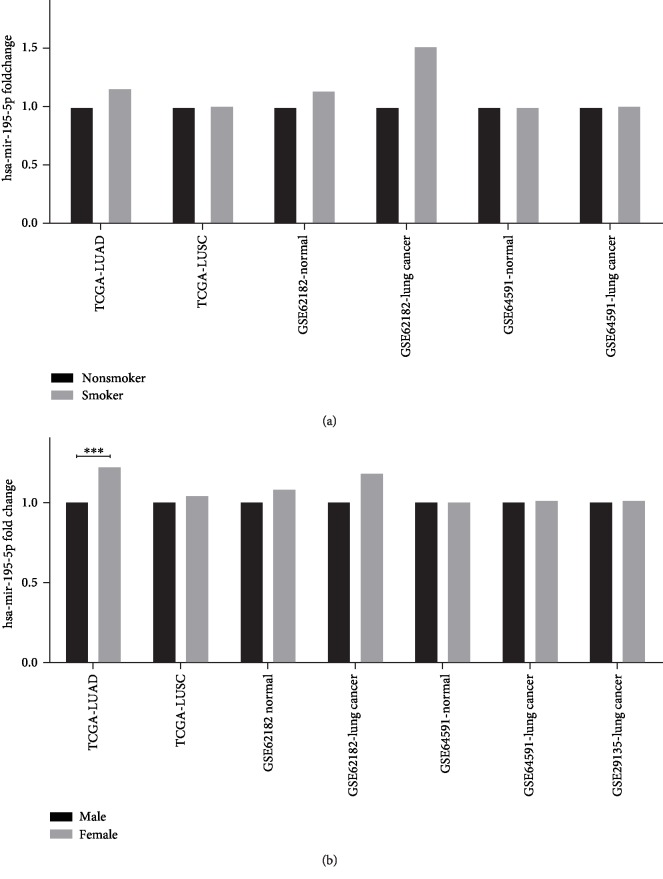
Expression of *hsa-miR-195-5p* in different sexes and smoking statuses among lung cancer and COPD patients. In most datasets, sex and smoking status of patients do not affect the expression of *hsa-miR-195-5p*. The expression of *hsa-miR-195-5p* is higher in females than in males.

Smoking status and sex information can be found in the TCGA-LUAD and TCGA-LUSC GSE62182 and GSE64591 datasets. The dataset GSE29135 only had information regarding the sex of patients. GSE62182 and GSE64591 datasets included both lung cancer patients and normal controls. Among lung cancer patients and normal controls, there were no differences between nonsmokers and smokers with regard to *hsa-miR-195-5p* expression (*p* > 0.05). All datasets showed no differences between males and females in *hsa-miR-195-5p* expression (*p* > 0.05), except for the TCGA-LUAD dataset. In the TCGA-LUAD dataset, females had a much higher *hsa-miR-195-5p* expression than males (*p* < 0.01).  Figure 2 shows the details of these analyses.

### 3.3. ROC Curve for Distinguishing Lung Cancer Patients, COPD Patients, and Normal Controls Using *hsa-miR-195-5p* Expression

**Figure 3:**
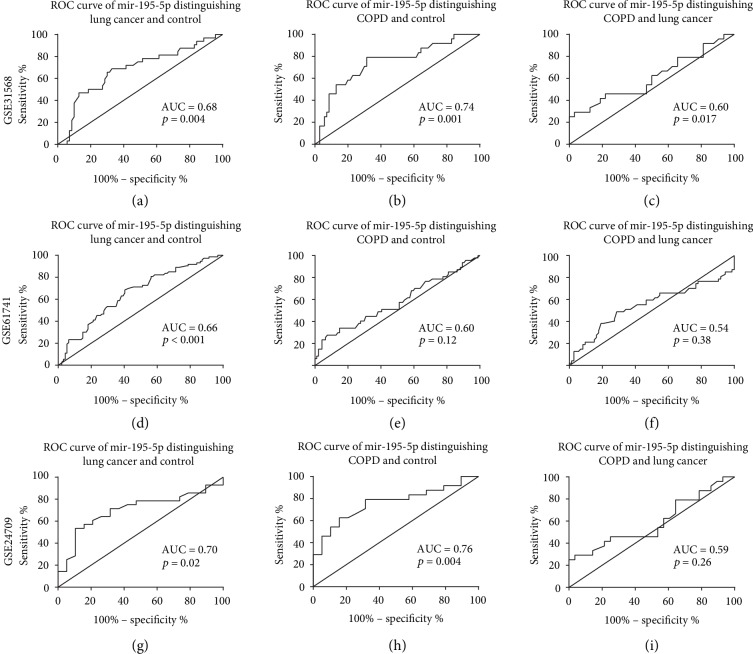
ROC curves of *hsa-miR-195-5p* to distinguish lung cancer patients, COPD patients, and normal controls. In all three datasets, *hsa-miR-195-5p* showed good performance in distinguishing between lung cancer or COPD patients and normal controls, but not between lung cancer and COPD patients.

ROC curves were performed to evaluate the ability of *hsa-miR-195-5p* to distinguish lung cancer and COPD patients from normal controls using the GSE24709, GSE61741, and GSE31568 datasets, which included lung cancer patients, COPD patients, and normal controls. Above all, *hsa-miR-195-5p* expression was able to distinguish well lung cancer patients and normal controls in three datasets (AUC > 0.65, *p* < 0.05). In distinguishing COPD patients and normal controls using *hsa-miR-195-5p* expression, the GSE31568 and GSE24709 datasets, but not the GSE61741 dataset, showed statistical significance (AUC > 0.70, *p* < 0.01). Using *hsa-miR-195-5p* expression to distinguish COPD and lung cancer patients, only the GSE61741 dataset showed significant differences (AUC = 0.60, *p* < 0.05), and another two datasets did not reach a significant difference (*p* > 0.05). The results from these analyses are shown in Figure 3.

### 3.4. Coexpression Network of *hsa-miR-195-5p* in the TCGA Lung Cancer Patients

**Figure 4:**
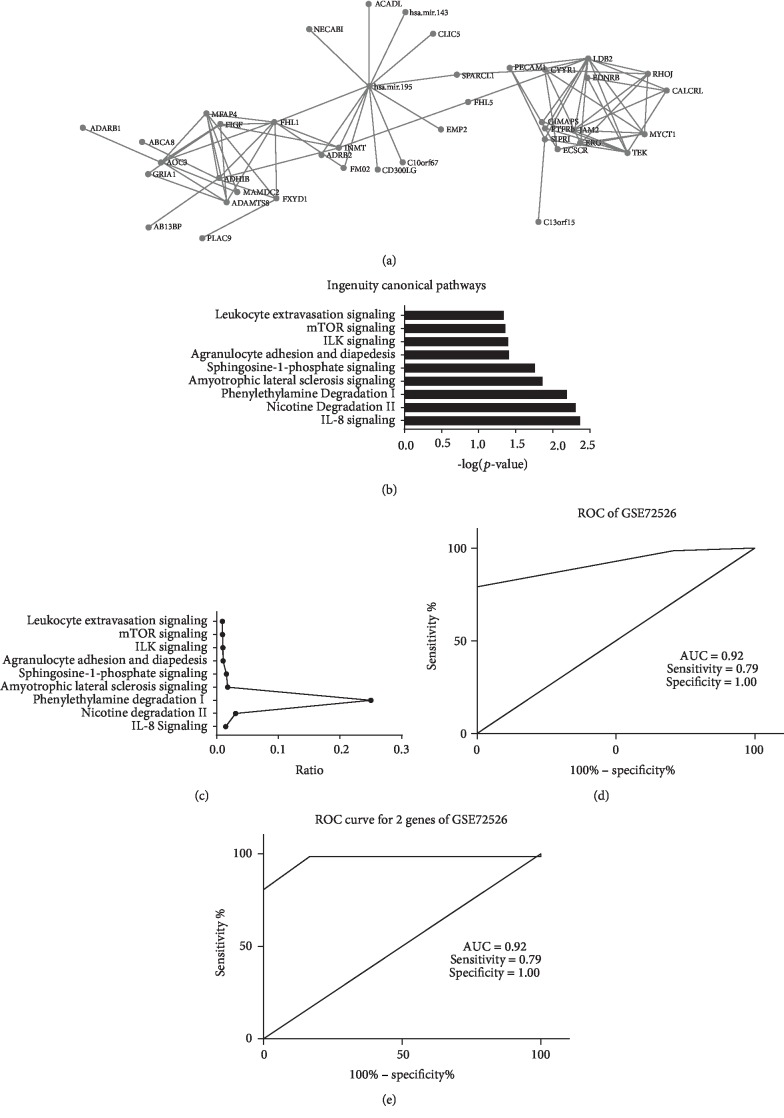
Coexpression network and Ingenuity Pathway Analysis of *hsa-miR-195* in the TCGA lung cancer patients The ROC curve of *hsa-miR-195* combined with *hsa-miR-143* to distinguish lung cancer patients and normal controls in another two datasets. (a) The network analysis shows another microRNA highly correlated with *hsa-miR-195*. (b, c) Ingenuity Pathway Analysis shows that the IL-8 signaling pathway was the most important pathway in this network. (d, e) The combination of *hsa-miR-195* and *hsa-miR-143* has a better performance in distinguishing lung cancer patients and normal controls compared with *hsa-miR-195* alone.

Depending on the TCGA lung cancer dataset, microRNA and mRNA expression data were merged. The genes directly linked to *hsa-miR-195-5p* with an *R*^2^ > 0.5 were selected as directly associated genes. In the network of these genes, which were not directly linked to *hsa-miR-195-5p*, the cutoff was *R*^2^ > 0.7. Thirteen genes were directly associated with *hsa-miR-195-5p* expression. IPA analysis was performed using directly associated genes together with another indirectly linked gene. The IL-8 signaling pathway was the most important pathway in this network and plays a key role in lung cancer patients (Figures 4(b) and 4(c)). Among thirteen genes, the microRNA hsa-miR-143 was selected as a candidate for further analyses; this miRNA has been widely reported to be associated with lung cancer. The results are shown in Figure 4(a).

### 3.5. ROC Curve for *hsa-miR-195-5p* Combined with *hsa-miR-143* to Distinguish Lung Cancer Patients and Normal Controls

The GSE72526 was a dataset using microRNA to predict *ALK*, *EGFR*, and *KRAS* statuses in lung cancer patients and to use *ALK*, *EGFR*, and *KRAS* as biomarkers to diagnose lung cancer. The sensitivity and specificity of this dataset were 0.64 and 1.00, respectively. In this dataset, *hsa-miR-195-5p* was used to predict lung cancer with a sensitivity and a specificity of 0.79 and 1.00, respectively (AUC = 0.92, *p* < 0.05). When *hsa-miR-195-5p* was combined with *hsa-miR-143*, the sensitivity and specificity were 0.99 and 0.83, respectively (AUC = 0.97, *p* < 0.05).  Table 1, Figure 4(d), and Figure 4(e) show the parameters of these analyses.

## 4. Discussion

For lung cancer screening, large research studies have been carried out. The Prostate, Lung, Colorectal and Ovarian Cancer Screening Trial (PLCO) is a cancer screening trial to determine whether a screening procedure reduces the mortality of PLCO cancers [26]. Based on this trial, researchers found that age, race or ethnicity, education, body mass index, COPD, personal history of cancer, family history of lung cancer, smoking status, smoking intensity, smoking duration, and smoking quit time influenced lung cancer morbidity [27]. This model can be used to assess the risk of lung cancer. The National Lung Screening Trial (NLST) is a multicentre, randomized clinical trial comparing low-dose helical computerized tomographic scanning (CT) with chest radiography in screening smokers for early detection of lung cancer [28]. A risk model was also made on the risk of lung cancer diagnosis. In this model, age, sex, race, smoking pack-years, emphysema on T0 CT, self-reported history of COPD, and family history of lung cancer were included [29]. Chronic obstructive pulmonary disease (COPD) is the fourth most common cause of death and smoking-related disorders [30]. COPD shares many risk factors with lung cancer, including smoking exposure, underweight, and low education [31, 32], and COPD itself is a risk factor for lung cancer [27]. Some lung cancer risk models considered COPD as a component [27].

In addition to these models comprised of classical phenotypes, some researchers discovered biomarkers to improve the prediction accuracy of the models [33]. The ITALUNG biomarker panel (IBP) combined with low-dose computed tomography achieved good performance for the identification of lung cancers at baseline screening, with a sensitivity of 90.0% and specificity of 89.0% [34]. A study suggested that a panel of four biomarkers composed of prolactin, *CRP*, *NY-ESO-1*, and *HGF* to screen for lung cancer. Combining this panel with sex, age, and smoking status, this analysis can achieve 86.96% sensitivity and 98.25% specificity for detecting lung cancer patients [35]. A study of a panel of transcript expressions of 14 antioxidants, DNA repair, and transcription factor genes in normal bronchial epithelial cells showed an AUC of 0.87 [36]. A lung cancer diagnostic panel consisting of *APOA1*, *CO4A*, *CRP*, *GSTP1*, and *SAMP* expression levels reached 95% sensitivity and 81% specificity [37]. Similarly, a panel of four biomarkers (*α*-2 macroglobulin, haptoglobin, ceruloplasmin, and hemopexin) was able to discriminate COPD patients and controls [38]. Sawa et al. reported that the frequency of the *PIK3CA* mutation increased in parallel with COPD severity, and the *PIK3CA* mutation is a genetic feature of patients with non-small-cell lung cancer (NSCLC) with COPD, regardless of age, smoking, pathological stage, and histology [39]. These biomarkers could improve the detection of lung cancer and COPD patients (the high-risk population for developing lung cancer).

MicroRNAs are a type of very short noncoding RNA. It is well known that miRNAs can bind to complementary sites in the 3′-untranslated region (UTR) of target mRNA, leading to posttranscriptional gene silencing. Many miRNAs have been discovered as biomarkers for the diagnosis of lung cancer and for stratifying lung cancer subtypes [40]. Jin et al. reported that *miR-181-5p*, *miR-30a-3p*, *miR-30e-3p*, *miR-361-5p*, *miR-10b-5p*, *miR-15b-5p*, and *miR-320b* can be used to NSCLC with an AUC value of 0.899 for detecting NSCLC [41]. Zhu et al. developed a signature containing 4 miRNAs, *miR-23b*, *miR-221*, *miR-148b*, and *miR-423-3p*, with an AUC of 0.885, and this signature may be considered as a biomarker for diagnosing lung cancer.

Bioinformatics is an appropriate approach for an initial discovery to identify biomarkers. Using bioinformatics methods, researchers found a series of microRNAs that can be used as diagnosis and prognosis biomarkers in tumors. The public databases such as GEO and TCGA and the prediction tools of mirDIP (http://ophid.utoronto.ca/mirDIP) and DIANA-mirPath (https://omictools.com/diana-mirpath-tool) are widely used in the researches of microRNAs. Based on a GEO dataset, Hafsi et al. found two microRNAs that targeted *YY1* mRNA in Burkitt's lymphoma using miRNA target prediction tools and Pearson correlation. The two microRNAs were related to the expression of *YY1* and downregulated in Burkitt's lymphoma [42]. Using two public available GEO datasets, Falzone et al. reported several microRNAs that were associated with the epithelial-mesenchymal transition pathway and NGAL/MMP-9 pathways in bladder cancer [43]; the author also found four microRNAs which were related to colorectal cancer through the mismatch repair pathway and other tumor signaling pathways [44]. The research group reported three microRNAs, which were associated with the prognosis in uveal melanomas [45]; in oral cancer, they identified 11 microRNAs with a potential diagnostic role and eight microRNAs associated with prognosis [46]. For the first time, they discovered a set of deregulated miRNAs in both glioblastoma and Alzheimer's disease [47].

In this study, we compared the expression of *hsa-miR-195-5p* between lung cancer patients, COPD patients, and normal controls. We found a lower expression of *hsa-miR-195-5p* in lung cancer and COPD patients. Using *hsa-miR-195-5p* as a biomarker to diagnose lung cancer, the AUC was 0.92, when combining *hsa-miR-195-5p* with the correlative microRNA *hsa-miR-143*, and the AUC was 0.97 for diagnosing lung cancer. Similarly, *hsa-miR-195-5p* has the ability to diagnose COPD, but the evidence was not strong enough to distinguishing lung cancer from COPD.

*hsa-miR-195-5p* is located at 17p13 with 87 bp in the genome. Our previous research has demonstrated the effect of *hsa-miR-195-5p* on the prognosis of NSCLC patients. *hsa-miR-195-5p* can suppress NSCLC by decreasing *CHEK1* expression [21]. *MiR-195* regulates the response of NSCLC to microtubule-targeting agents (MTAs) by targeting *CHEK1* [48]. In addition to lung cancer, *miR-195* suppresses colon cancer proliferation and metastasis [49], inhibits tumour growth and angiogenesis in breast cancer [50], and is associated with the chemotherapy sensitivity of cisplatin and the clinical prognosis in gastric cancer [51]. In the studies of Falzone, *hsa-miR-195-5p* is one of the 16 microRNAs, which are downregulated in oral cancer [45]; in colorectal cancer, *hsa-miR-195-5p* is also downregulated and directly related to colorectal cancer through some cancer pathways [5]. *hsa-miR-143* is located at 5q32 with 106 bp in the genome. *MiR-143* can suppress gastric cancer cell migration and metastasis by inhibiting *MYO6* and EMT [52]; it can also regulate the proliferation and migration of osteosarcoma through *MAPK7* [53]. *hsa-miR-143-5p* is upregulated in uveal melanomas [45] and bladder cancer [43] in bioinformatics studies. The ability of *hsa-miR-195-5p* to diagnose lung cancer and to find a high-risk population has not been reported.

This study has some limitations. We did not have enough clinical samples to confirm the results. The clinical information of the datasets is not complete; therefore, we could not analyze the subtypes of lung cancer and COPD. In addition, we could not adjust the relevant factors properly. The pathway and functional analysis remained at the sample level and was only a little exploration for mechanistic research.

## 5. Conclusions

Early diagnosis and detection of lung cancer are effective strategies for prevention and treatment. *hsa-miR-195-5p* has a good performance as a biomarker to diagnose lung cancer. *hsa-miR-195-5p* may contribute to the diagnosis of lung cancer and the detection of its high-risk population.

## Figures and Tables

**Table 1:** Statistical parameters of *has-mir-195-5p* combined with *has-mir-143* to distinguish lung cancer and normal.

Parameter	*has-miR-195-5p*	*has-miR-195-5p* & *has-miR-143*
Sensitivity	0.79	0.99
Specificity	1.00	0.83
False positive rate	0.00	0.17
False negative rate	0.21	0.02
Accuracy	0.84	0.95
Kappa	0.62	0.85
AUC	0.92	0.97

## Data Availability

The datasets generated and/or analyzed during the current study are available in the GEO repository, https://www.ncbi.nlm.nih.gov/gds, and TCGA repository, https://portal.gdc.cancer.gov/.

## Abbreviations

COPD: Chronic obstructive pulmonary disease
NSCLC: Non-small-cell lung cancer
GEO: Gene expression omnibus
TCGA: The cancer genome atlas
LUAD: Lung adenocarcinoma
LUSC: Lung squamous cell carcinoma
ROC: Receiver operating characteristic curves
AUC: Area under the curve
NLST: The national lung screening trial
PLCO: The prostate, lung, colorectal, and ovarian cancer screening trial.

## Supplementary Materials

Supplementary Materials Table S1: summarized information of the databases. This table summarized the details of the database used in this article, including the brand of the platform, the type of platform, the type of technology, the type of experiment, the number of samples, the type of samples, and other relevant information of samples.
