# Supplementary material for: MicroRNA Biomarker hsa-miR-195-5p for Detecting the Risk of Lung Cancer
Source: Int J Genomics. 2020 Jan 2;2020:7415909. doi: 10.1155/2020/7415909 (PMC6961786; doi:10.1155/2020/7415909)
Supplement: Supplementary Materials — Table S1: summarized information of the databases. This table summarized the details of the database used in this article, including the brand of the platform, the type of platform, the type of technology, the type of experiment, the number of samples, the type of samples, and other relevant information of samples. [file 7415909.f1.docx]

**Table S1. Summary of the datasets**

| **Dataset ID** | **Brand of the platform** | **Platform** | **Technology type** | **Experiment type** | **Number of tumor** | **Number of COPD** | **Number of control** | **Type of samples** | **Has sex information** | **Has smoking information** |
| --- | --- | --- | --- | --- | --- | --- | --- | --- | --- | --- |
| GSE15008 | CapitalBio | GPL8176 National Engineering Research Center mammalian microRNA microarray | Spotted oligonucleotide | Non-coding RNA profiling by array | 187 | 0 | 188 | Fesh cancer tissue and adjacent normal tissue from patient | No | No |
| GSE62182 | Illumina Inc. | GPL11154Illumina HiSeq 2000 (Homo sapiens) | High-throughput sequencing | Non-coding RNA profiling by high throughput sequencing | 94 | 0 | 94 | Fresh-frozen tumor and matched normal tissues | Yes | Yes |
| GSE64591 | Life technologies | GPL18942 Applied Biosystems Taqman Low Density Array Human microRNA Card A+B Set v3.0 | RT-PCR | Non-coding RNA profiling by array | 100 | 0 | 100 | Peripheral blood | Yes | Yes |
| GSE49881 | Invitrogen | GPL15892 NCode Human miRNA microarray V3 | Spotted oligonucleotide | Non-coding RNA profiling by array | 0 | 41 | 16 | Fesh whole lung tissue | Yes | Yes |
| GSE31568 | Febit biomed | GPL9040 febit Homo Sapiens miRBase 13.0 | In situ oligonucleotide | Non-coding RNA profiling by array | 32 | 24 | 70 | Blood | No | No |
| GSE61741 | Febit biomed | GPL9040 febit Homo Sapiens miRBase 13.0 | In situ oligonucleotide | Non-coding RNA profiling by array | 72 | 47 | 94 | Blood | No | No |
| GSE24709 | Febit biomed | GPL9040 febit Homo Sapiens miRBase 13.0 | In situ oligonucleotide | Non-coding RNA profiling by array | 28 | 24 | 19 | Blood | No | No |
| GSE17681 | Febit biomed | GPL9040 febit Homo Sapiens miRBase 13.0 | In situ oligonucleotide | Non-coding RNA profiling by array | 17 | 0 | 19 | Blood | No | No |
| GSE29135 | Illumina Inc. | GPL8179 Illumina Human v2 MicroRNA expression beadchip | Oligonucleotide beads | Non-coding RNA profiling by array | 387 | 0 | 0 | Formalin-fixed and paraffin-embedded tumor tissues | Yes | No |
| GSE72526 | Nanostring | GPL20275 NanoString nCounter Human miRNA Expression Assay v2 [mRNA ID] | Other | Non-coding RNA profiling by array | 67 | 0 | 18 | Formalin-fixed, paraffin-embedded tissues | No | No |
| TCGA-LUAD | Illumina Inc. | Illumina HiSeq RNASeqV2 and Illumina HiSeq miRNASeq | NA | miRNA-Seq and RNA-Seq | 517 | 0 | 59 | Primary Solid Tumor and Solid Tissue Normal | Yes | Yes |
| TCGA-LUSC | Illumina Inc. | Illumina HiSeq RNASeqV3 and Illumina HiSeq miRNASeq | NA | miRNA-Seq and RNA-Seq | 501 | 0 | 51 | Primary Solid Tumor and Solid Tissue Normal | Yes | Yes |
